# Partial and Total Annoyance Due to Road Traffic Noise Combined with Aircraft or Railway Noise: Structural Equation Analysis

**DOI:** 10.3390/ijerph14121478

**Published:** 2017-11-30

**Authors:** Laure-Anne Gille, Catherine Marquis-Favre, Kin-Che Lam

**Affiliations:** 1 Univ Lyon, ENTPE, Laboratoire Genie Civil et Batiment, 3 rue Maurice Audin, F-69518 Vaulx-en-Velin, France; laureanne_gillechambo@yahoo.com; 2 Department of Geography & Resource Management, The Chinese University of Hong-Kong, Hong Kong, China; kinchelam@cuhk.edu.hk

**Keywords:** transportation noise, noise sensitivity, noise annoyance, structural equation modeling, combined noise sources

## Abstract

Structural equation modeling was used to analyze partial and total in situ annoyance in combined transportation noise situations. A psychophysical total annoyance model and a perceptual total annoyance model were proposed. Results show a high contribution of *Noise exposure* and *Noise sensitivity* to *Noise annoyance*, as well as a causal relationship between noise annoyance and lower *Dwelling satisfaction.* Moreover, the *Visibility of noise source* may increase noise annoyance, even when the visible noise source is different from the annoying source under study. With regards to total annoyance due to road traffic noise combined with railway or aircraft noise, even though in both situations road traffic noise may be considered background noise and the other noise source event noise, the contribution of road traffic noise to the models is greater than railway noise and smaller than aircraft noise. This finding may be explained by the difference in sound pressure levels between these two types of combined exposures or by the aircraft noise level, which may also indicate the city in which the respondents live. Finally, the results highlight the importance of sample size and variable distribution in the database, as different results can be observed depending on the sample or variables considered.

## 1. Introduction

Noise exposure is a major environmental concern for industrial country citizens (e.g., [1]). Indeed, unlike other environmental pollutants, noise levels are still increasing [1] due to urbanization and traffic growth. To manage exposure to transportation noise, it is therefore necessary to better understand the various factors influencing noise annoyance and how these factors interact with each other.

It has been shown that noise annoyance is influenced by different acoustical factors, such as the noise level (e.g., [2,3,4,5,6,7,8,9]) and the type of noise source (e.g., [10,11]). Socio-acoustic surveys have shown that numerous individual characteristics or reactions may also influence annoyance due to transportation noise. For example, individual socio-economic characteristics (e.g., [2,3,7,8,9]), noise sensitivity (e.g., [2,3,6,8,9,12]), dwelling characteristics (e.g., [2,7]), emotional and physical health (e.g., [5,7]), activity disturbance or sleep disturbance (e.g., [3,4,5,6,7,8,9]), impression of the living area (e.g., [3,6]), or relation to the noise source or to the noise source authority (e.g., [4,5,8]), may influence noise annoyance. Some individual characteristics or reactions may be influenced by other individual characteristics or reactions and by acoustical factors. For example, sleep disturbance, which impacts noise annoyance, is itself influenced by noise exposure (e.g., [8]). Given the complex relationships that exist between the various variables involved, several authors have used structural equation modeling to analyze noise annoyance using survey data (e.g., [2,3,4,5,6,7,8,9]). This statistical technique based on linear regression makes it possible to study wide database samples and to model the direct and indirect effects of multiple factors on a dependent variable.

Furthermore, some authors have shown that relationships between factors may vary according to cultural differences [9] and according to the nature of the dominant source in combined noise exposure situations [13]. In 2012, the French Ministry of Ecology ordered a socio-acoustic survey in order to study annoyance due to combined transportation noise (i.e., road traffic noise combined with railway traffic and/or aircraft noise, railway traffic noise combined with aircraft noise) in France. The objectives of the present paper are: (i) to explore the role of non-acoustical factors influencing noise annoyance; (ii) to study partial noise annoyance due to road traffic, railway and aircraft noise in combined exposure using structural equation modeling; and (iii) to study total noise annoyance due to combined exposure.

## 2. Presentation of the French Socio-Acoustic Survey

This study is based on the survey carried out by Ecotiere et al. [14] performed in 8 French cities exposed to different combinations of transportation noise (i.e., road and rail; road and air, rail and air; and road, rail and air transportation noise). The survey did not include cities exposed to only one transportation noise. Respondents were aged between 18 and 80 and had been living permanently in their dwelling for at least one year. They were interviewed face-to-face about their personal impressions. A total of 823 people were successfully interviewed. Interviews were held in French.

### 2.1. Questionnaire

The questionnaire included questions concerning:

- neighborhood, living environment, and housing;
- global environment;
- noise from the different sources under study, considered separately (i.e., road traffic, railway traffic, and aircraft noise, depending on the city of residence) and annoyance due to each noise source (hereafter referred to as “partial annoyance”, as annoyance due to each noise source is evaluated in the presence of another transportation noise source);
- overall noise resulting from the combined noise sources under study (i.e., road and rail, road and air, rail and air, as well as road, rail and air transportation noise sources, and annoyance due to these combined noise sources (hereafter referred to as “total annoyance”);
- non-acoustical factors related to the respondent (e.g., noise sensitivity).

The questions concerning partial and total noise annoyances complied with ISO/TS 15666 technical specification recommendations for French language [15] (e.g., “Thinking back over the last 12 months, when you are here at home, how do you rate on a scale from 0 to 10 how annoyed you are by road traffic noise?”; and “Thinking back over the last 12 months, when you are here at home, how do you rate on a scale from 0 to 10 how annoyed you are by road traffic noise and the railway traffic noise together?”). Respondents were asked to give an annoyance rating to the specified noise source(s) on a continuous scale ranging from “0” to “10”, comprised of 11 evenly spaced numerical labels and two verbal labels at both ends (“not at all” and “extremely”). The questions and scale concerning noise sensitivity were built on the same format ([14]).

### 2.2. Noise Exposure in the Surveyed Cities

Noise annoyance was studied in eight cities exposed to combined transportation noise exposure. The exposure of each respondent was determined using strategic noise maps drawn in 2012 for each studied city by technical services under contract with the French government, using European Directive 2002/49/CE guidelines. In particular, such noise maps were established for each noise source in isolation and displayed noise exposure in terms of the $L_{den}$ index, that is to say, for an average day. Table 1 describes the noise exposure both in terms of range of $L_{den}$—day–evening–night level—and in terms of sample size.

### 2.3. Respondents of the Survey

Table 2 shows the characteristics of the French study population. The average age of the study population is approximately 46 years. Relatively more women than men participated in the study. Some residents live since only one year in their dwelling, whereas some others live in since very long time (maximal value: 77 years). The survey presents therefore variability in the interviewed inhabitants in terms of socio-demographic characteristics.

## 3. Results

In this section, partial noise annoyance due to road traffic, aircraft, or railway noise, and total noise annoyance due to combined exposures are modeled using structural equation modeling. Structural equation modeling and the variables introduced in the annoyance models are first described. Partial annoyance is then modeled. Finally, total noise annoyance models are derived from the partial annoyance models.

### 3.1. Presentation and Computation of Structural Equation Modeling

Structural equation modeling is a statistical technique that enables the study of complex relationships among variables, where some variables may be unobservable. As noise annoyance can be influenced by numerous variables that can also interact with each other, structural equation modeling is a relevant tool applied here to the French survey database.

Structural equation modeling follows a confirmatory approach. The first step is to propose a model for relationships between the variables. Structural equation modeling analysis will then show if the model is consistent with the data. However, this statistical technique is based on large samples. In order to use structural equation modeling, the minimum sample size should be more than 200, or 5–20 times the number of parameters to be estimated, whichever is larger [16].

Considering the sample size for each combined situation (Table 1), two situations of combined noise can be modeled: road traffic noise combined with aircraft noise, and road traffic noise combined with railway noise.

In the following discussion, manifest variables (i.e., measured variables) will be shown as rectangles whereas latent variables (i.e., unobserved variables) will be represented as ovals. Endogenous variables should also be distinguished from exogenous variables: an exogenous variable is a variable that influences other variables, whereas an endogenous variable is influenced by other variables (and can also influence other variables).

Structural equation models are calculated using SEPATH of Statistica. Standardized coefficients are estimated using the correlation matrix of the variables. Goodness-of-fit (GFI) is used to assess the adjustment quality of the model. Empirically, the model is validated if GFI value is superior to 0.9 ([17]).

### 3.2. Variables Considered in the Annoyance Models

To propose an annoyance model, findings of previous literature dealing with structural equation modeling of noise annoyance have been used to select relevant variables and relevant relationships between variables [2,3,4,5,6,7,8,9,12]. Variables introduced in annoyance model are:

- single *Noise exposure*, given in $L_{den}$ (range given in Table 1);
- *Noise sensitivity*, self-rated during the survey on a continuous scale ranging from 0 to 10 (Section 2.1);
- *Noise annoyance*, rated on a continuous scale ranging from 0 to 10 (Section 2.1);
- *Disturbance due to noise*, recoded as a yes/no question using the six following questions: “When you are here at home, does outdoor noise cause you to: sometimes interrupt your conversation/increase TV sound/wake up/be disturbed when you are reading/be disturbed when you are relaxing or resting/not use your garden or your balcony?” (This recoded variable was introduced as a manifest variable in the various models. Introducing *Disturbance due to noise* as a latent variable influenced by six manifest variables (one per question) was not possible as the model would have had too many degrees of freedom for the number of available observations.);
- *Dwelling satisfaction*, self-rated on a categorical five-point scale ranging 1–5;
- *Visibility of a main road* and *Visibility of railway track*, respectively recoded as a yes/no question using the following question for each room: “What does this room overlook?” (The visibility of aircraft was not introduced in the models as no dwellings were facing the runway or the airport, whereas some dwellings were facing a main road or a railway track).

The recoded variables (*Disturbance due to noise*, *Visibility of a main road* and *Visibility of railway track*) are presented in Table A1 in Appendix in terms of percentages of No/Yes answers, depending on the combined noise exposure database considered in the structural equation modeling.

### 3.3. Partial Noise Annoyance

**Figure 1:**
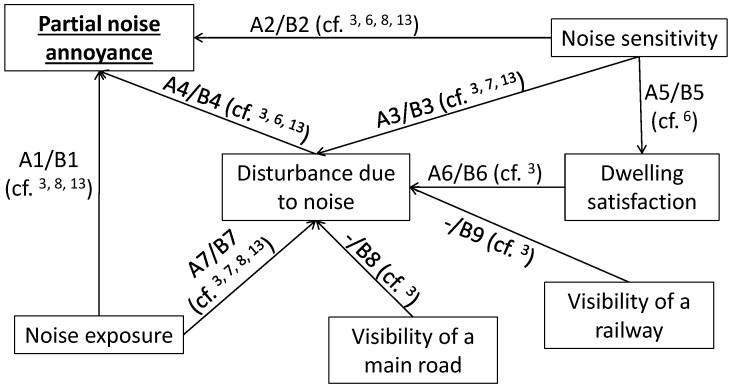
Structural equation model of partial noise annoyance without (Model A) and with (Model B) *Visibility of noise source*. A1 to A7 represent standardized coefficients in Model A, while B1 to B9 represent standardized coefficients in Model B.

Different partial annoyance models were tested using the previously described variables. The two more complex and significant models were kept: model without *Visibility of noise source* (“Model A”) and model with *Visibility of noise source* (“Model B”). The two models were kept because: (i) both models were found significant; (ii) Model B is more complex than Model A; and (iii) Model B highlights complex relationships between a given noise source and the partial annoyance due to another noise source. Figure 1 represents these structural equation models. Relationships between variables were selected according to literature findings. Some examples of relevant literature references are given for each relationship. For example, *Disturbance due to noise* is influenced by *Noise exposure*, *Noise sensitivity*, *Dwelling satisfaction*, and in Model B by *Visibility of railway track* and *Visibility of a main road*. *Disturbance due to noise* influences partial *Noise annoyance*. In the partial noise annoyance models, all variables are manifest. The variables *Noise exposure*, *Visibility of a main road*, *Visibility of railway track*, and *Noise sensitivity* are exogenous variables, as they are not influenced by other variables. The other variables are endogenous variables.

#### 3.3.1. Road Traffic Noise Annoyance

Table 3 gives the standardized coefficients for Models A and B calculated for partial noise annoyance due to road traffic noise. These models were calculated based on different database samples: (i) the whole database of respondents exposed to road traffic noise (i.e., 702 respondents, Table 1); (ii) cities exposed to road traffic noise combined with aircraft noise (i.e., 212 respondents); and (iii) cities exposed to road traffic noise combined with railway noise (i.e., 301 respondents). Table 4 gives the direct, indirect and total effects on noise annoyance of each variable (The indirect effect of a variable on *Noise annoyance* is measured using the effect of a variable on *Noise annoyance* through an other variable ([18]). For example, *Noise sensitivity* has an indirect effect on *Noise annoyance* through *Dwelling satisfaction* and *Disturbance due to noise*. The value of the indirect effect of *Noise sensitivity* on *Noise annoyance* is equal to (A5×A6+A3)×A4). The contribution of exogenous variables (i.e., variables that are not influenced by other variables) to the models is also given in percentage (Exogenous variable contribution is calculated by dividing the total effect of the variable by the sum of the total effect of all the exogenous variables. For example, in Model A, using the whole database, *Noise exposure* contribution equals to 0.162/(0.162 + 0.372) = 30.34%.)

The results displayed in Table 4 for Model A show that *Noise sensitivity* influences *Noise annoyance* more than *Noise exposure* does. Furthermore, results are of the same order of magnitude whether for the whole database or for solely cities exposed to road traffic noise combined with aircraft noise. In cities exposed to road traffic noise combined with railway noise, the difference between the total effect of *Noise sensitivity* and the total effect of *Noise exposure* is smaller than what is observed in the other samples of the database. Finally, it is noted that *Dwelling satisfaction* is the only variable which does not increase along with other variables. Indeed, coefficients A5 and A6 are negative, which means that the more respondents are sensitive to noise, the less they are satisfied with their dwelling, and the more they are disturbed by noise.

The results displayed in Table 4 for Model B show that *Noise sensitivity* has the strongest effect on *Noise annoyance*, followed by *Noise exposure*. The main difference between the various database samples is the influence of the *Visibility of railway track*. In the sample of cities exposed to road traffic noise combined with aircraft noise, the *Visibility of railway track* has no influence on *Noise annoyance*. However, in the whole database sample and in the sample of cities exposed to road traffic noise combined with railway noise, the *Visibility of railway track* has an effect on *Noise annoyance* due to road traffic noise, with the same order of magnitude (respectively, 8.08% and 9.91%) than the *Visibility of a main road* (respectively, 8.59% and 6%). The differing contributions of the *Visibility of railway track* variable on *Noise annoyance* is due to differing values of this variable within each sample (Table A1 in Appendix). In fact, in cities exposed to road traffic noise combined with aircraft noise, there are no railway tracks visible from the respondents’ dwellings: this variable has therefore no variance for this database sample and cannot be introduced in the annoyance model. This also explains why the GFI value for the whole database is very close to significance levels but not significant: the variance of the *Visibility of railway track* may not be sufficient to warrant the introduction of this variable in a significant model.

In Model B, it is also noted that *Noise exposure* has an influence on *Disturbance due to noise* only when considering cities exposed to road traffic noise combined with aircraft noise. This result is surprising as this relationship was significant for all database samples in Model A. Two explanations stand to explain this difference between Models A and B:

- The introduction of two new variables (*Visibility of a main road* and *Visibility of railway track*) with their significant link to *Disturbance due to noise* (B8 and B9) makes the link between *Noise exposure* and *Disturbance due to noise* (B7) non-significant;
- Since the *Visibility of a main road* is already partially factored in road traffic *Noise exposure* (dwellings facing a main road are exposed to higher road traffic noise levels), the introduction of this variable decreases the influence of *Noise exposure* on *Disturbance due to noise*.

However, these explanations cannot account for the difference observed between the various survey samples. Such difference may be due to the *Visibility of railway track*, because where *Visibility of railway track* influences *Disturbance due to noise*, *Noise exposure* does not and vice versa.

#### 3.3.2. Aircraft Noise Annoyance

As was the case with road traffic noise, structural equation modeling leads to two types of models (Models A and B) for partial annoyance due to aircraft noise. Table 5 gives the standardized coefficients for Models A and B calculated for partial noise annoyance due to aircraft noise, based on the various samples of the database. Table 6 gives the direct, indirect and total effects of each variable on noise annoyance.

The results displayed in Table 6 for Model A show that the contribution of *Noise sensitivity* and of *Noise exposure* are of the same order of magnitude. It appears that the difference between the contribution of the two variables is weaker than in the case of annoyance due to road traffic noise (Table 4).

The results displayed in Table 6 for Model B show that the *Visibility of railway track* has no effect on aircraft *Noise annoyance*, as opposed to the *Visibility of a main road*. The contribution of the variables on *Noise annoyance* depends on the sample used, as indicated by the values obtained (Table A1 in Appendix). For the whole database sample, it appears that *Noise exposure* contributes more than *Noise sensitivity*, whereas in the sample of cities exposed to road traffic noise combined with aircraft noise, it is the opposite. This difference may be explained by the variance in *Noise exposure*. Indeed, in cities exposed to combined road traffic and aircraft noises, aircraft *Noise exposure* may vary between 42 dB(A) in Paray-Vieille-Poste and (52–54) dB(A) in Saint-Brice-sous-Forêt, whereas in the whole database, aircraft noise exposure ranges continuously from 42 dB(A) to 65.3 dB(A) (Table 1). Thus, in view of the difference in aircraft noise exposure levels, it appears that *Noise exposure* to aircraft is also an indication of the city the respondents live in and the airport they are exposed to.

#### 3.3.3. Railway Noise Annoyance

Structural equation modeling leads to two types of models (A and B) for partial annoyance due to railway noise, without and with *Visibility of noise source*. Table 7 gives the standardized coefficients for Models A and B calculated for partial noise annoyance due to railway noise, based on the various database samples. Table 8 gives the direct, indirect and total effects of each variable on noise annoyance.

The results displayed in Table 8 for Model A show that *Noise sensitivity* contributes to railway *Noise annoyance* more than *Noise exposure* does, as was the case for road traffic noise annoyance (Section 3.3.1). However, contrary to Model A for road traffic or aircraft noise annoyance, *Noise exposure* has no effect on *Disturbance due to noise*.

The results displayed in Table 8 for Model B show that *Noise sensitivity* has the strongest effect on *Noise annoyance*, followed by *Noise exposure*. The *Visibility of a main road* influences *Disturbance due to noise*, only in cities exposed to road traffic noise combined with railway noise, perhaps because of the variability of this variable in the whole database.

### 3.4. Total Noise Annoyance

Depending on the data sample size, total annoyance can be studied in only two combined noise situations:

- total noise annoyance due to road traffic noise combined with aircraft noise;
- total noise annoyance due to road traffic noise combined with railway noise.

It was not possible to study total annoyance using structural equation modeling when aircraft noise was combined with railway noise or when road traffic noise was combined with combined aircraft and railway noises.

Two kinds of total annoyance models were drawn:

- a psychophysical model (Model “psy”): total noise annoyance is influenced by *Noise exposure* to each noise source;
- a perceptual model (Model “per”): total noise annoyance is influenced by *Partial noise annoyance* due to each noise source.

In the psychophysical model, *Combined noise exposure* is introduced as a latent variable, influenced by the single *Noise exposure* manifest variables. This enables us to highlight the separate contributions of each noise source to the total annoyance model.

In both kinds of total annoyance models, two variations were studied: without (Model psy-A or per-A) and with (Model psy-B or per-B) *Visibility of a main road/railway track*.

**Figure 2:**
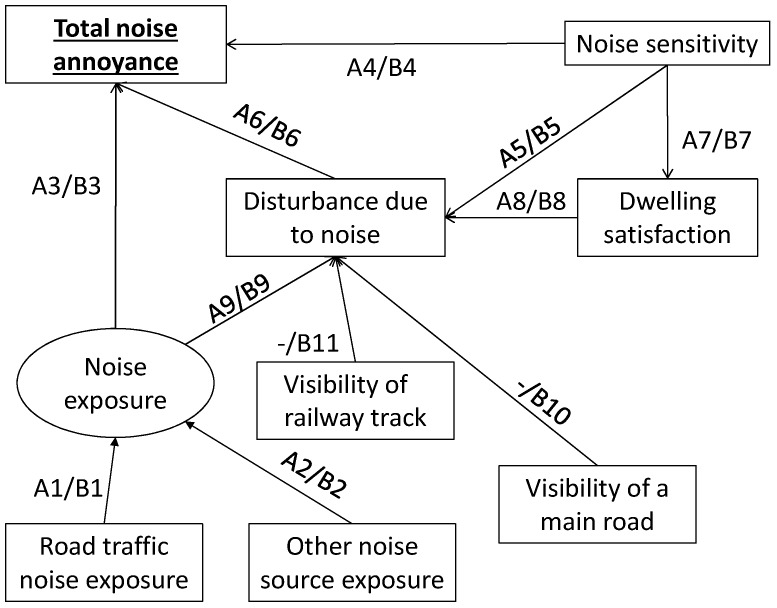
Psychophysical structural equation model of total noise annoyance without and with *Visibility of noise source*. A1 to A9 represent the standardized coefficients in Model psy-A, whereas B1 to B11 represent the standardized coefficients in Model psy-B. The variable *Combined noise exposure* is represented in an oval as it is a latent variable influenced by the *Noise exposure* manifest variables.

**Figure 3:**
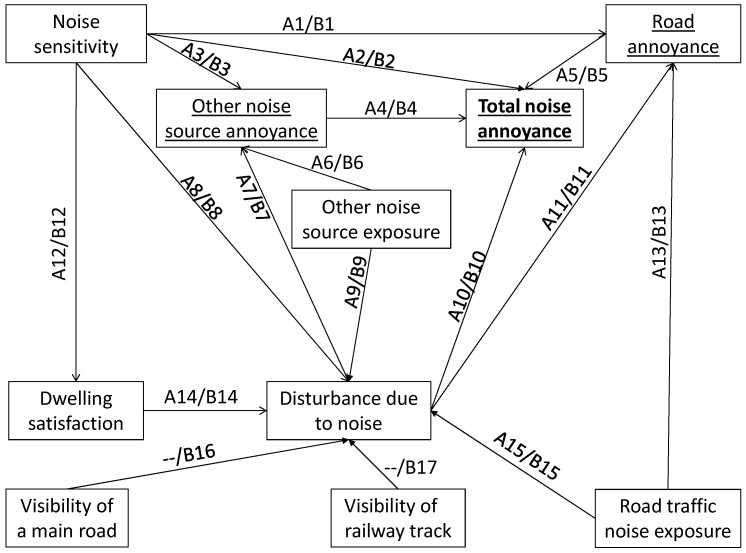
Perceptual structural equation model of total noise annoyance without and with *Visibility of noise source.* A1 to A15 represent the standardized coefficients in Model per-A, whereas B1 to B17 represent the standardized coefficients in Model per-B.

Figure 2 and Figure 3 represent these structural equation models.

The models were calculated for total annoyance due to road traffic noise combined with aircraft noise (Section 3.4.1), and for total annoyance due to road traffic noise combined with railway noise (Section 3.4.2). Standardized coefficients of the various models are given in Table 9. Both direct and indirect effects of each variable on noise annoyance are summarized in Table 10 for psychophysical total annoyance models and in Table 11 for perceptual total annoyance models.

#### 3.4.1. Road Traffic Noise Combined with Aircraft Noise

In psychophysical models (Table 10), the variable with the highest contribution is *Noise sensitivity*, followed by *Aircraft noise exposure* and *Road traffic noise exposure*. The *Visibility of railway track* has no effect on *Disturbance due to noise*. This is due to the variance of this variable in cities exposed to road traffic noise combined with aircraft noise (as mentioned in Section 3.3.1). The contribution of *Aircraft noise exposure* is the same in Models psy-A and psy-B. On the other hand, the introduction of the *Visibility of a main road* variable decreases the contribution of *Noise sensitivity* and of *Road traffic noise exposure*.

In perceptual models (Table 11), it is noted that the contributions of variables in Model per-A are almost the same as in Model psy-A. The perceptual model (with the introduction of *Partial noise annoyances* in the model) does not result in variations in the contributions of variables. On the other hand, Model per-B (with *Visibility of a main road*) shows contribution levels of *Noise sensitivity* and of *Aircraft noise exposure* similar to those in Model psy-B, which is not the case for *Road traffic noise exposure* (smaller contribution) and *Visibility of a main road* (slightly higher contribution).

#### 3.4.2. Road Traffic Noise Combined with Railway Noise

In psychophysical models (Table 10), the variable with the highest contribution is *Noise sensitivity*, followed by *Road traffic noise exposure*, and finally *Railway noise exposure*. The opposite was observed for road traffic noise combined with aircraft noise. In Model psy-B, both *Visibility of a main road* and *Visibility of railway track* have an influence on *Disturbance due to noise*.

In perceptual models (Table 11), the contribution of *Railway noise exposure* is almost the same in Models psy-A and psy-B, which is not the case for *Road traffic noise exposure* but it appears that it is higher in perceptual models than in psychophysical models. In addition, it is noted that, for Model per-B, the GFI value is not significant, probably because of the high number of variables and relationships that are taken into account.

## 4. Discussion

The French survey data were used to observe the relationships between partial or total annoyance due to transportation noise, acoustical factors (such as *Noise exposure*), and non-acoustical factors (such as *Noise sensitivity*, *Disturbance due to noise*, *Visibility of noise source*, or *Dwelling satisfaction*).

In line with the findings of other studies dealing with noise annoyance due to transportation noise (e.g., [8,19]), this study shows that *Noise sensitivity* is generally the main contributor in most annoyance models (Table 4, Table 6, Table 8, Table 10 and Table 11); however, it is not the main contributor in models for partial annoyance due to aircraft noise. Considering this result, it would be relevant to introduce noise sensitivity in noise annoyance models to predict noise annoyance both for single noise exposure and for combined noise exposures, in the context of noise management, as already proposed in other studies [10,19].

As previously mentioned, the exception of partial annoyance due to aircraft noise may be explained by the distribution pattern of the aircraft *Noise exposure* variable across the database. Indeed, when considering cities exposed to road traffic noise combined with aircraft noise, the aircraft *Noise exposure* variable is an indication of the city in which the respondents live and the airport to which they are exposed (Table 1). In this particular case, this variable is therefore both acoustical and non-acoustical. Furthermore, for the particular case of aircraft noise, some authors (e.g., [20]) have shown that noise annoyance is influenced by a non-acoustical factor that is specific to each airport, namely the community tolerance level (CTL). This may explain why aircraft *Noise exposure*, as an acoustical and non-acoustical variable, contributes so much to the model for cities exposed to road traffic noise combined with aircraft noise.

Furthermore, since other studies (e.g., [21,22]) indicated that *Visibility of noise source* increases *Noise annoyance*, this variable was introduced in the models used in the present study. Results show that *Visibility of a main road* or *Visibility of railway track* may indirectly influence partial and total annoyance due to transportation noise, even when the visible noise source is not the one that causes noise annoyance. For example, *Noise annoyance* due to road traffic is influenced by the *Visibility of a main road* and the *Visibility of railway track*. This finding is in contrast with Izumi and Yano [3], where the variable “Open facade facing on to road” had no significant influence on annoyance due to road traffic noise. The discrepancy between the two studies may be explained by cultural differences—in this paper, the survey took place in France, whereas Izumi and Yano [3] studied annoyance in Japan— and also by the fact that all the respondents in the French survey were exposed to combined noise sources. It would therefore appear that in France, one way to reduce noise annoyance due to transportation noise is to make the noise source not visible from the dwelling (at least not from main rooms such as the living room). This is in agreement with the recommendation to preserve access to quietness at the least exposed facade of the dwelling, i.e., to maintain a noticeable noise level difference between the most and the least exposed facades (e.g., [23]).

With regards to total annoyance due to combined noises, one interesting point is that the noise source with the main contribution to the model differs according to the combined noise situation. In the case of road traffic noise combined with aircraft noise, the main source is aircraft noise, whereas in the case of road traffic noise combined with railway noise, the main source is road traffic noise (Table 10 and Table 11). These findings are in contrast with studies where road traffic noise is background noise, and railway/aircraft noise is event noise. In actual fact, such studies analyze highway road traffic noise (e.g., [24]). In such cases, railway noise is more annoying than road traffic noise because of its intermittent character compared to the more permanent character of highway road traffic noise. In the current study, road traffic noise is urban road traffic noise, with singular events due to vehicle acceleration and deceleration, and to specific vehicles such as powered two-wheelers. The current results are in line with studies pointing out that aircraft noise is more annoying than road traffic noise, the latter being more annoying than railway noise (e.g., [11]). Another explanation for the annoying noise source ranking of the current study can be found in Lam et al. [13]. Studying road traffic noise combined with railway noise, Lam et al. [13] found that noise disturbance is attributed to railway noise rather than road traffic noise when the road traffic noise level is higher than the railway noise level. They hypothesized that in this kind of situation, the high level of road traffic noise may sensitize the respondents to the peaks of railway noise events, which is not the case when the railway noise level is higher than the road traffic noise level. With regards to the noise levels of the French survey (Table 1), in most road traffic noise combined with aircraft noise situations, aircraft noise levels are lower than road traffic noise levels, and so the ranking of these two noise sources in terms of annoyance may be better explained by this sensitization effect.

Finally, the current study highlights existing limitations in structural equation modeling terms of sample size effect and collinear variable effect, as is the case with other statistical modeling techniques (e.g., multiple regression). The first step in structural equation modeling is to propose a model of relationships between different variables. Therefore, the results obtained depend on the variables that are introduced and on the models that are proposed. Furthermore, the more complex the model is, the more numerous must observations be in order to properly assess the model. This is why results may differ in Models A and B. Model B is a more complex model with more variables. The introduction of new variables may result in variable redundancy, or the model might be too complex to be significantly evaluated on the considered sample. Moreover, in this particular study, the results themselves are sample-dependent. Differing results are observed for annoyance due to transportation noise depending on the database sample used. For example, the *Visibility of railway track* does not influence *Noise annoyance* due to road traffic noise when solely considering cities exposed to road traffic noise combined with aircraft noise, whereas it does influence *Noise annoyance* when considering other database samples (Section 3.3.1). This is explained by the values of the variable across the database (Table A1 in Appendix).

## 5. Conclusions

A socio-acoustic survey that was conducted in eight French cities to study both partial and total noise annoyances in combined transportation noise situations was used to analyze the influence of acoustical and non-acoustical factors on noise annoyance due to transportation noise, using structural equation modeling. For total annoyance due to combined noises, a psychophysical model and a perceptual model were proposed. Results show the high contribution of *Noise exposure* and *Noise sensitivity*. Other non-acoustical factors that influence both partial and total annoyances are *Disturbance due to noise*, *Dwelling satisfaction*, and *Visibility of noise source*. The relationships between variables and the contribution of the variables to the model depend on the noise source considered. Furthermore, different results may be observed depending on the database sub-sample considered, which highlights the important role played in structural equation modeling by the distribution of the variables across the sample and by the size of the sample. Moreover, the analysis of the different transportation noise source contributions shows that aircraft noise has a higher influence on total annoyance than road traffic noise, and that in turn road traffic noise has a higher influence on total annoyance than railway noise.

## Figures and Tables

**Table 1:** Noise exposure given in $L_{den}$ (dB(A)) per noise source and sample size of areas in cities under study during the survey ([14]). Road: Road traffic, Rail: Railway and Air: Aircraft. ${}^{1}$: city exposed to Orly airport noise, ${}^{2}$: city exposed to Roissy-Charles-de-Gaulle airport noise.

Combined Exposures	City	$L_{\mathrm{den}}$ Road Traffic dB(A)	$L_{\mathrm{den}}$ Railway dB(A)	$L_{\mathrm{den}}$ Aircraft dB(A)	Sample Size	Total Sample Size
Road and Rail	Bourg Les Valence	58.0 to 80.8	58.1 to 77.4	no exposure	82	301
	Caluire	55.6 to 78.0	45.6 to 82.2		79	
	Lyon 6	48.4 to 74.2	40.6 to 83.6		140	
Road and Air	Paray Vieille Poste ${}^{1}$	49.9 to 77.9	no exposure	42.0	153	212
	St Brice ss Foret ${}^{2}$	53.7 to 67.5		52.0 to 54.0	59	
Rail and Air	Goussainville ${}^{2}$	below 55	52.0 to 72.0	49.0 to 60.0	96	121
	Villeneuve Le Roi ${}^{1}$	not studied	54.4 to 71.9	54.0 to 65.3	25	
Road, Rail and Air	Villeneuve St Georges ${}^{1}$	42.3 to 79.3	43.2 to 80.8	44.7 to 62.8	189	189
	Sample size	702	611	522		

**Table 2:** Characteristics of the French study population.

	N (%)	Mean ± SD (Min–Max)
**Gender**		
Male	48.48%	
Female	51.52%	
**Age**		46.01 ± 16.89 (18–80)
**Length of residence**		
in the dwelling		13.4 ± 14.07 (1–77)
in the neighborhood		15.99 ± 15.85 (1–77)
**Occupation**		
Working	55.89%	
(1) Regular hours	38.52%	
Shift hours	17.37%	
(2) At home	6.56%	
Not at home	49.33%	
Non-working	44.11%	
Retired	22.72%	
Student	4.86%	
Unemployed	7.41%	
Housewife	6.68%	
Disabled	1.22%	

**Table 3:** Standardized coefficients used in models of the partial annoyance due to road traffic noise. Models are based on different samples of the French in situ survey database. ns: non-significant.

Model	Coeff.	Whole Database	Road Traffic with Aircraft	Road Traffic with Railway
A	A1	0.124	0.131	0.245
	A2	0.265	0.314	0.258
	A3	0.286	0.27	0.365
	A4	0.329	0.374	0.347
	A5	−0.237	−0.236	−0.287
	A6	−0.162	−0.144	−0.114
	A7	0.116	0.183	0.103
	GFI	0.989	0.975	0.995
B	B1	0.125	0.132	0.248
	B2	0.266	0.317	0.261
	B3	0.278	0.25	0.356
	B4	0.329	0.371	0.349
	B5	−0.237	−0.236	−0.287
	B6	−0.165	−0.129	−0.116
	B7	ns	0.144	ns
	B8	0.155	0.22	0.131
	B9	0.145	ns	0.218
	GFI	0.888	0.948	0.94

**Table 4:** Direct (dir.), indirect (ind.) and total (tot.) effects of all variables affecting noise annoyance due to road traffic noise in Models A and B. Contributions of exogenous variables are given in percentage between brackets.

Model	Variable	Whole Database			Road Traffic with Aircraft			Road Traffic with Railway		
		dir.	ind.	tot.	dir.	ind.	tot.	dir.	ind.	tot.
A	*Noise exposure*	0.124	0.038	0.162	0.131	0.068	0.199	0.245	0.036	0.281
		(30.34%)			(31.74%)			(41.51%)		
	*Noise sensitivity*	0.265	0.107	0.372	0.314	0.114	0.428	0.258	0.138	0.396
		(69.66%)			(68.26%)			(58.49%)		
	*Disturbance due to noise*	0.329	–	0.329	0.374	–	0.374	0.347	–	0.347
	*Dwelling satisfaction*	–	−0.053	−0.053	–	−0.054	−0.054	–	−0.040	−0.040
B	*Noise exposure*	0.125	–	0.125	0.132	0.053	0.185	0.248	–	0.248
		(21.04%)			(26.89%)			(32.33%)		
	*Noise sensitivity*	0.266	0.104	0.370	0.317	0.104	0.421	0.261	0.136	0.397
		(62.29%)			(61.19%)			(51.76%)		
	*Visibility of a main road*	–	0.051	0.051	–	0.082	0.082	–	0.046	0.046
		(8.59%)			(11.92%)			(6.00%)		
	*Visibility of railway track*	–	0.048	0.048	–	–	–	–	0.076	0.076
		(8.08%)			–			(9.91%)		
	*Disturbance due to noise*	0.329	–	0.329	0.371	–	0.371	0.349	–	0.349
	*Dwelling satisfaction*	–	−0.054	−0.054	–	−0.048	−0.048	–	−0.040	−0.040

**Table 5:** Standardized coefficients used in models of the partial annoyance due to aircraft noise. Models are based on different samples of the French in situ survey database.

Model	Coeff.	Whole Database	Road Traffic with Aircraft
A	A1	0.380	0.301
	A2	0.297	0.26
	A3	0.225	0.245
	A4	0.271	0.249
	A5	−0.229	−0.236
	A6	−0.121	−0.154
	A7	0.362	0.213
	GFI	0.975	0.986
B	B1	0.38	0.132
	B2	0.297	0.317
	B3	0.219	0.25
	B4	0.271	0.371
	B5	−0.229	−0.236
	B6	−0.11	−0.129
	B7	0.37	0.144
	B8	0.142	0.22
	B9	ns	ns
	GFI	0.974	0.948

**Table 6:** Direct (dir.), indirect (ind.) and total (tot.) effects of all variables affecting noise annoyance due to aircraft noise in Models A and B. Contributions of exogenous variables are given in percentage between brackets.

Model	Variable	Whole Database			Road Traffic with Aircraft		
		dir.	ind.	tot.	dir.	ind.	tot.
A	*Noise exposure*	0.380	0.098	0.478	0.301	0.053	0.354
		(56.70%)			(51.75%)		
	*Noise sensitivity*	0.297	0.068	0.365	0.260	0.070	0.330
		(43.30%)			(48.25%)		
	*Disturbance due to noise*	0.271	–	0.271	0.249	–	0.249
	*Dwelling satisfaction*	–	0.033	0.033	–	0.038	0.038
B	*Noise exposure*	0.380	0.100	0.480	0.132	0.053	0.185
		(54.48%)			(26.89%)		
	*Noise sensitivity*	0.297	0.066	0.363	0.317	0.104	0.421
		(41.20%)			(61.19%)		
	*Visibility of a main road*	–	0.038	0.038	–	0.082	0.082
		(4.31%)			(11.92%)		
	*Visibility of railway track*	–	–	–	–	–	–
	*Disturbance due to noise*	0.271	–	0.271	0.371	–	0.371
	*Dwelling satisfaction*	–	0.030	0.030	–	0.048	0.048

**Table 7:** Standardized coefficients used in models of the partial annoyance due to railway noise. Models are based on different samples of the French in situ survey database.

Model	Coeff.	Whole Database	Road Traffic with Railway
A	A1	0.232	0.256
	A2	0.265	0.29
	A3	0.302	0.368
	A4	0.261	0.338
	A5	−0.245	−0.287
	A6	−0.142	−0.119
	A7	ns	ns
	GFI	0.998	0.986
B	B1	0.232	0.248
	B2	0.265	0.261
	B3	0.295	0.356
	B4	0.261	0.349
	B5	−0.245	−0.287
	B6	−0.145	−0.116
	B7	ns	ns
	B8	ns	0.131
	B9	0.096	0.218
	GFI	0.938	0.94

**Table 8:** Direct (dir.), indirect (ind.) and total (tot.) effects of all variables affecting noise annoyance due to railway noise in Models A and B. Contributions of exogenous variables are given in percentage between brackets.

Model	Variable	Whole Database			Road Traffic with Railway		
		dir.	ind.	tot.	dir.	ind.	tot.
A	*Noise exposure*	0.232	–	0.232	0.256	–	0.256
		(39.66%)			(37.54%)		
	*Noise sensitivity*	0.265	0.088	0.353	0.290	0.136	0.426
		(60.34%)			(62.46%)		
	*Disturbance due to noise*	0.261	–	0.261	0.338	–	0.338
	*Dwelling satisfaction*	–	−0.037	−0.037	–	0.040	0.040
B	*Noise exposure*	0.232	–	0.232	0.248	–	0.248
		(38.16%)			(32.33%)		
	*Noise sensitivity*	0.265	0.086	0.351	0.261	0.136	0.397
		(57.73%)			(51.76%)		
	*Visibility of a main road*	–	–	–	–	0.046	0.046
		–			(6.00%)		
	*Visibility of railway track*	–	0.025	0.025	–	0.076	0.076
		(4.11%)			(9.91%)		
	*Disturbance due to noise*	0.261	–	0.261	0.349	–	0.349
	*Dwelling satisfaction*	–	−0.038	−0.038	–	−0.040	−0.040

**Table 9:** Standardized coefficients for both psychophysical and perceptual models of total annoyance due to combined noises, without and with *Visibility of noise source*.

Model	Coeff.	Road Traffic with Aircraft	Road Traffic with Railway	Model	Coeff.	Road Traffic with Aircraft	Road Traffic with Railway
psy-A	A1	0.397	1.000	psy-B	B1	0.330	1.000
	A2	0.595	0.241		B2	0.714	0.241
	A3	0.219	0.189		B3	0.196	0.189
	A4	0.356	0.293		B4	0.351	0.294
	A5	0.245	0.368		B5	0.217	0.356
	A6	0.370	0.480		B6	0.390	0.478
	A7	−0.236	−0.287		B7	−0.236	−0.287
	A8	−0.145	−0.119		B8	−0.134	−0.116
	A9	0.374	–		B9	0.322	–
					B10	0.247	0.131
					B11		0.218
	GFI	0.971	0.984		GFI	0.956	0.922
per-A	A1	0.316	0.258	per-B	B1	0.320	0.261
	A2	0.090			B2	0.090	
	A3	0.261	0.291		B3	0.260	0.291
	A4	0.479	0.419		B4	0.483	0.422
	A5	0.445	0.493		B5	0.442	0.492
	A6	0.302	0.256		B6	0.302	0.257
	A7	0.249	0.337		B7	0.248	0.336
	A8	0.243	0.365		B8	0.214	0.356
	A9	0.182	–		B9	0.229	–
	A10	0.152	0.200		B10	0.153	0.200
	A11	0.373	0.347		B11	0.376	0.349
	A12	−0.236	−0.287		B12	−0.236	−0.287
	A13	0.132	0.245		B13	0.134	0.248
	A14	−0.161	−0.114		B14	−0.139	−0.116
	A15	0.146	0.103		B15		
					B16	0.260	0.131
					B17		0.218
	GFI	0.931	0.922		GFI	0.915	0.883

**Table 10:** Direct (dir.), indirect (ind.) and total (tot.) effects of all variables affecting *Noise annoyance* due to combined noises in psychophysical Models psy-A and psy-B. Contributions of exogenous variables are given in percentage between brackets.

Variable	Road Traffic with Aircraft						Road Traffic with Railway					
	psy-A			psy-B			psy-A			psy-B		
	dir.	ind.	tot.	dir.	ind.	tot.	dir.	ind.	tot.	dir.	ind.	tot.
*Road traffic noise exposure*	–	0.142	0.142	–	0.106	0.106	–	0.189	0.189	–	0.189	0.189
	(17.43%)			(12.06%)			(26.23%)			(21.44%)		
*Other noise source exposure*	–	0.213	0.213	–	0.230	0.230	–	0.046	0.046	–	0.046	0.046
	(26.13%)			(26.09%)			(6.32%)			(5.17%)		
*Noise sensitivity*	0.356	0.103	0.459	0.351	0.097	0.448	0.293	0.193	0.486	0.294	0.186	0.480
	(56.44%)			(50.90%)			(67.45%)			(54.46%)		
*Disturbance due to noise*	0.370	–	0.370	0.390	–	0.390	0.480	–	0.480	0.478	–	0.478
*Dwelling satisfaction*	–	−0.054	−0.054	–	−0.052	−0.052	–	−0.057	−0.057	–	−0.055	−0.055
*Visibility of a main road*	Not in the model			–	0.096	0.096	Not in the model			–	0.063	0.063
				(10.95%)						(7.10%)		
*Visibility of railway track*	Not in the model			–	–	–	Not in the model			–	0.104	0.104
				–						(11.82%)		

**Table 11:** Direct (dir.), indirect (ind.) and total (tot.) effects of all variables affecting *Noise annoyance* due to combined noises in perceptual Models per-A and per-B. Contributions of exogenous variables are given in percentage between brackets.

Variable	Road Traffic with Aircraft						Road Traffic with Railway					
	per-A			per-B			per-A			per-B		
	dir.	ind.	tot.	dir.	ind.	tot.	dir.	ind.	tot.	dir.	ind.	tot.
*Road traffic noise exposure*	–	0.123	0.123	–	0.059	0.059	–	0.174	0.174	–	0.122	0.122
	(14.85%)			(6.69%)			(23.65%)			(14.17%)		
*Other noise source exposure*	–	0.224	0.224	–	0.246	0.246	–	0.107	0.107	–	0.108	0.108
	(27.17%)			(27.84%)			(14.62%)			(12.60%)		
*Noise sensitivity*	0.090	0.389	0.479	0.090	0.375	0.465	–	0.453	0.453	–	0.451	0.451
	(57.98%)			(52.58%)			(61.73%)			(52.41%)		
*Disturbance due to noise*	0.152	0.285	0.437	0.153	0.286	0.439	0.200	0.312	0.512	0.200	0.314	0.514
*Dwelling satisfaction*	–	−0.070	−0.070	–	−0.061	−0.061	–	−0.058	−0.058	–	−0.060	−0.060
*Road annoyance*	0.445	–	0.445	0.442	–	0.442	0.493	–	0.493	0.492	–	0.492
*Other noise source annoyance*	0.479	–	0.479	0.483	–	0.483	0.419	–	0.419	0.422	–	0.422
*Visibility of a main road*	Not in the model			–	0.114	0.114	Not in the model			–	0.067	0.067
				12.89%						(7.81%)		
*Visibility of railway track*	Not in the model			–	–	–	Not in the model			–	0.112	0.112
				–						(13.00%)		

## Appendix

**Table A1:** Values of Yes-No variables in terms of percentages.

No/Yes (Percentages)	Disturbance Due to Noise	Visibility of a Main Road	Visibility of Railway Track
Whole database for road traffic noise	41.17%/58.83%	63.11%/36.89%	83.62%/16.38%
Whole database for aircraft noise	39.66%/60.34%	81.23%/18.77%	92.34%/7.66%
Whole database for railway noise	30.44%/69.56%	62.36%/37.64%	76.60%/23.40%
Road traffic combined with aircraft	62.74%/37.26%	80.66%/19.34%	100%/0%
Road traffic combined with railway	37.21%/62.79%	42.52%/57.48%	65.78%/34.22%
